# Optimization of the functional characteristics, pasting and rheological properties of pearl millet-based composite flour

**DOI:** 10.1016/j.heliyon.2017.e00240

**Published:** 2017-02-04

**Authors:** Olugbenga Olufemi Awolu

**Affiliations:** Department of Food Science and Technology, Federal University of Technology, Akure, Nigeria

**Keywords:** Food Science

## Abstract

Optimisation of composite flour comprising pearl millet, kidney beans and tigernut with xanthan gum was evaluated for rheological evaluations. The functional properties of the composite flour were optimized using optimal design of response surface methodology. The optimum blends, defined as blends with overall best functional characteristics were run 3 (75.956% pearl millet, 17.692% kidney beans, 6.352% tigernut flours), run 7 (85.000% pearl millet, 10.000% kidney beans, 5.000% tigernut flours) and run 13 (75.000% pearl millet, 20.000% kidney beans, 5.000% tigernut flours). The pasting characteristics and rheological evaluation of the optimized blends were further evaluated in rapid visco units (RVU). Run 7 had the overall best pasting characteristics; peak viscosity (462 RVU), trough (442 RVU), breakdown viscosity (20 RVU), final viscosity (975 RVU), setback (533 RVU), peak time (5.47 min) and pasting temperature (89.60 °C). These values were found to be better than several composite flours consisting mixture of wheat and non-wheat crops. In addition, the rheological characteristics (measured by Mixolab) showed that run 7 is the best in terms of dough stability, swelling, water absorption and shelf stability. Composite flour with 85% pearl millet flour in addition to kidney beans and tigernut flours could therefore serve as a viable alternative to 100% wheat flour in bread production.

## 1 Introduction

The use of cereals, tubers with or without legumes and fibres as viable sources of functional composite flours keeps on increasing (Bamigbola et al., 2016; Awolu et al., 2016b; Awolu et al., 2016a). Partial utilization or non-utilization of wheat in flour production are meant to mitigate gluten-related celiac disease, diversify raw materials for flour production especially using local and nutritionally rich crops, and reduce high cost of importing wheat by developing countries. Cereals (rice, amaranth, tigernut), tubers (cocoyam, sweet potatoes), legumes (soybeans, African oil bean, bambara groundnut, kersting’s groundnut) and fibres sources (rice bran, brewer’s spent grains) have been utilized as credible sources of composite flours (Bamigbola et al., 2016; Awolu et al., 2016b; Awolu et al., 2016a; Awolu et al., 2015).

Pearl millet is a species of millet widely grown in Nigeria; Nigeria being the world’s third largest producer after India and Niger (FAO, 1995). Millets generally have been discovered to be rich in dietary fibre, minerals, phytochemicals (especially phenolic compounds) and vitamins which make them to be health promoting (Saleh et al., 2013). Millets have been incorporated into wheat in the production of bread, biscuits, and ready-to-eat snacks (Saha et al., 2011).

Kidney beans is an underutilized tropical legume rich in protein, ash, soluble and insoluble fibres, and linoleic acid (Manonmani et al., 2014). It has been used to complement cereals and wheat in the production of composite flours rich in protein. Tigernut on the other hand is also an underutilized crop rich in calcium, magnesium, iron, dietary fibre, protein and carbohydrates (Oladele and Aina, 2007). It has been widely used in food formulations and composite flours production (Awolu et al., 2016b; Ade-Omowaye et al., 200811).

Mixolab measures dough development behaviour during mixing and heating, thereby enabling determination of contribution of protein and starch to dough rheology (Vizitiu and Danciu, 2011; Rosell et al., 2007). It helps expression of consistency (torque) of dough rheology in SI unit (Nm) distinguishing it from brabender unit which is an arbitrary unit. Mixolab has been shown to have advantages over farinograph (Vizitiu and Danciu, 2011).

Currently, there is little or no work on the pearl millet-kidney beans composite flour. In addition to utilizing pearl millet-kidney bean, this work further added tigernut flour and carry out the optimization of the functional, pasting and rheological properties of pearl millet-based composite flour as viable alternative to wheat flour in bread production.

## 2 Materials and methods

### 2.1 Materials

Pearl Millet *(pennisetum glaucuml)* was obtained from Mile 12 market, Lagos State; Xanthan gum was obtained from Payless Chemical Store, Ojota, Lagos, Nigeria; Tigernut *(Cyperus esculentus)* was obtained from Iddo market, Lagos; Kidney bean *(Phaseolus vulgans)* was purchased at Iddo market, Lagos State;

### 2.2 Preparation of pearl millet flour

The method of Jideani (2005) was used. Pearl millet seeds (1 Kg) was sorted and thoroughly washed using warm (65 °C) water. It was later oven-dried (thermostated oven, Model MC-1959 K, China) at 50 °C for 24 h, milled using locally fabricated attrition mill and passed through 200 μm sieve in order to obtain fine pearl millet flour, stored in a sealed plastic container at room temperature for further processing.

### 2.3 Preparation of tigernut flour

Tigernut flour was produced using the method of Adejuyitan et al., 2009. Tigernut (250 g) was sorted, washed thoroughly using warm (65 °C) water and oven-dried (thermostated oven, Model MC-1959 K, China) at 70 °C for 5 h. The clean, dried tigernut was milled in a locally fabricated attrition mill (Manufactured in Nigeria) and passed through a 200 μm sieve to obtain fine tigernut flour which was stored at room temperature in a sealed plastic container for further processing.

### 2.4 Preparation of kidney beans flour

The method of Giami and Bakebain (1992) was adopted. Kidney beans seeds (250 g) were washed, soaked in water for 30 min, dehulled, boiled at 100 °C for 30 min, oven-dried (thermostated oven, Model MC-1959 K, China) at 65 °C for 6 h, milled using locally fabricated attrition mill (made in Nigeria) and made to pass through 200 μm sieve. It was subsequently stored in a sealed plastic container at room temperature for further processing.

### 2.5 Experimental design

The experimental design was carried out using optimal mixture design of response surface methodology (Design Expert, 8.0.3.1 trial version). The independent variables were pearl millet flour (75.00–85.00 g/100 g), kidney beans flour (10.00–20.00 g/100 g), and tigernut flour (5.00–10.00 g/100 g). Xanthan gum (0.5 g/100 g) was added to all the samples in order to enhance its viscoelastic characteristics. The dependent variables are the functional characteristics.

### 2.6 Determination of bulk density

The method described by Oladele and Aina (2007) was used for the determination of bulk density. Flour sample (50 g) was weighed into 100 ml measuring cylinder. The measuring cylinder was then tapped continuously until a constant volume was obtained. Bulk density (g/cm^3^) was calculated using the formula:

$$\text{Bulk}\text{Density}\text{g}/\text{ml}=\frac{\text{Weight}\text{of}\text{sample}}{\text{Volume}\text{of}\text{sample}\text{after}\text{tapping}}$$

### 2.7 Determination of dispersibility

This was determined by the method described by Kulkarni et al. (1991). The flour sample (10 g) was weighed into a graduated cylinder. Water was added to the make up to 100 ml mark. It was shaken vigorously, and allowed to stand for 3 h. The volume of settled particles was recorded.

### 2.8 Determination of water absorption capacity

Water absorption capacity (WAC) of sample was determined using the method outlined by Diniz and Martin (1997) with some modifications. About 0.5 g of the sample was dissolved with 10 ml of distilled water in centrifuge tubes and vortexed for 30 s. The dispersions were allowed to stand at room temperature for 30 min, centrifuged at 3000 rpm for 25 min. The supernatant was filtered with Whatman No 1 filter paper and the volume retrieved was accurately measured. The difference between initial volumes of distilled water added to the sample and the volume obtained after filtration was determined. The results were reported as mL of water absorbed per gram of sample.

$$WaterAbsorptionCapacity=\frac{Amountofwaterabsorbed}{Weightofsample}$$

### 2.9 Determination of oil absorption capacity

Oil absorption capacity (OAC) was determined using the method of AOAC (2005). About 1 g of the sample (W_0_) was weighed into pre-weighed 15 ml centrifuge tubes and thoroughly mixed with 10 ml (V_1_) of refined pure groundnut oil using vortex mixer. Samples were allowed to stand for 30 min. The sample-oil mixture was centrifuged at 3000 rpm for 20 min. Immediately after centrifugation, the supernatant was carefully poured into a 10 ml graduated cylinder, and the volume was recorded (V_2_). Oil absorption capacity (milliliter of oil per gram of sample) was calculated.

$$OilAbsorptionCapacity=\frac{V_{1}-V_{2}}{W_{0}}$$

### 2.10 Determination of foaming capacity

Foaming capacity (FC) was determined in triplicate using the method described by Narayana and Narsinga (1992). Concentration of 1% of the sample was prepared in deionized water and adjusted to pH 7.4 with 1.0 N NaOH and 1.0 N HCl. A volume of 100 ml (V_1_) of concentrate suspension was blended for 3 min using a high speed blender, poured into a 250 ml graduated cylinder, and the volume of foam (V_F_) was immediately recorded. Foaming capacity was calculated using;

$$FC=\left(\frac{V_{F}}{V_{1}}\right)\times 100$$

### 2.11 Determination of swelling index

The method of Ukpabi and Ndimele (1990) was used. The swelling index (SI) of the samples was determined by putting 25 g of each sample in a 210 ml measuring cylinder. Distilled water (150 ml) was added and allowed to stand for four hours before observing the level of swelling. The swelling index was calculated.

$$SI=\frac{volumeaftersoaking-volumebeforesoaking}{weightofsample}$$

### 2.12 Determination of swelling capacity

The gel obtained from swelling index was used in calculating swelling capacity (SC) thus:

$$\%SC=\frac{weightofwetgel}{weightofsample}\times 100$$

### 2.13 Determination of pasting properties

The pasting properties of the samples were assessed using the Rapid Visco-Analyser (Model RVA series 4; Newport Scientific Pty Ltd., Warriewood, Australia). A 3 g sample was dispersed in an aluminum canister containing 25 ml of distilled water. The samples were tested according to Standard Profile 1, where the flour-water suspension was held at 50 °C for 1 min and then heated to 95 °C, held for 10 min, and then cooled to 50 °C and held for another 2 min. The starch viscosity parameters measured were pasting temperature, peak viscosity, breakdown viscosity, final viscosity, trough viscosity setback viscosity and peak time. The results were expressed as RVU for all of the parameters with the exception of pasting temperature, which is expressed in °C.

### 2.14 Determination of rheological behaviour of dough

Mixolab (‘Chopin + ’, Chopin Technologies, France) was used according to standard methods (AACC 54–60.01). Mixolab consisted of heating/cooling cycle after constant mixing at 80 rpm. The cooling temperature was 50 °C while the maximum temperature was 90 °C at one heating/cooling cycle. The analyses were carried out at constant water absorption according to Pastukhov and Dogan (2014). The dough characteristics were determined when mixing at constant temperature and during one heating/cooling cycle. Based on the result of pasting characteristics, two runs will be chosen for rheological analysis.

### 2.15 Statistical analysis

Statistical analyses were carried out using the Response Surface Methodology so as to fit the quadratic polynomial equation generated by the Design-Expert software version 8.0.3.1 (Stat-Ease Inc., Minneapolis, U.S.A). In order to correlate the response variable to the independent variables, multiple regressions was used to fit the coefficient of the polynomial model of the response. The quality of the fit of model was evaluated using analysis of variance (ANOVA).

## 3 Results and discussion

### 3.1 Functional properties of the composite flour

The results of the functional properties of the composite flour are presented in Table 1. The bulk density ranged from 0.69 to 1.03 g/ml. Increase in pearl millet content accounted for the high bulk density observed. The ANOVA showed that the model (Special quartic) and the model terms did not significantly (p ≥ 0.05) affect the bulk density, meaning that though there was an increase brought about by addition of pearl millet, the increase was not significant (p ≥ 0.05). The R^2^ and adjusted R^2^ values of 0.6170 and 0.1792 respectively obtained buttressed the assertion. Bamigbola et al. (2016) obtained bulk density of 0.70 g/ml for 100% wheat flour, and 0.63 and 0.90 g/ml for composite flours consisting 65.66–77% wheat flour, 29% plantain flour, 5.33% tigernut flour and 77% wheat flour, 20% plantain flour, 3% tigernut flour respectively. It has been observed that high bulk density is a desirable characteristic for the packaging of food materials of high nutrient contents (Hassan et al., 2013). Low density, however, has been found to be useful in the formulating complementary foods (Akpata and Akubor, 1999).

**Fig. 1:**
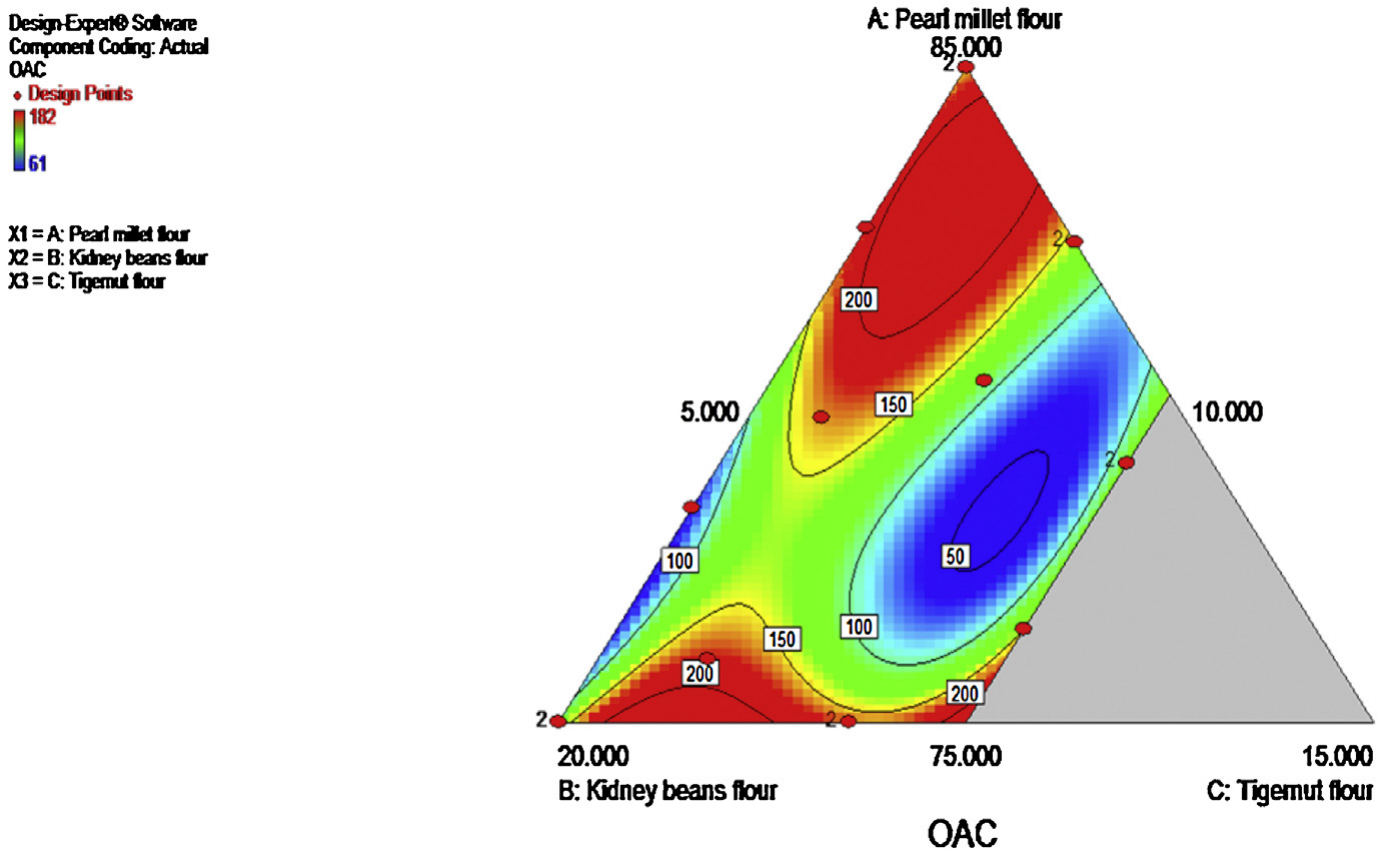
Contour plot showing the effect of pearl millet, kidney beans and tigernut composite flours on dispersibility.

The dispersibility of the composite flour ranged from 71–80%. Kidney beans significantly (p ≤ 0.05) increase the dispersibility. The R^2^ and adjusted R^2^ values were 0.9230 and 0.8075 respectively, while the p-value (0.9733) the for lack of fit was not significant (p ≥ 0.05). A high adjusted R^2^ is an indication of less variations between the dependent and independent variables, hence, it is desirable (Awolu et al., 2013; Awolu and Layokun, 2013; Awolu et al., 2015). In addition, a non-significant lack of fit is required (Awolu et al., 2013; Awolu and Layokun, 2013). The p-value for lack of (0.9733) is an indication that the model is highly fit (97.33%). The contour plot (Fig. 1) shows that kidney beans had the highest contribution to the dispersibility. The ANOVA showed that the model (Cubic) and model terms (Linear mixture, AB, BC(B-C) were significant (p ≤ 0.05). Eke-Ejiofor (2015) reported that high dispersibility enhanced better reconstitution of starch in water to give fine and constituent paste.

**Fig. 2:**
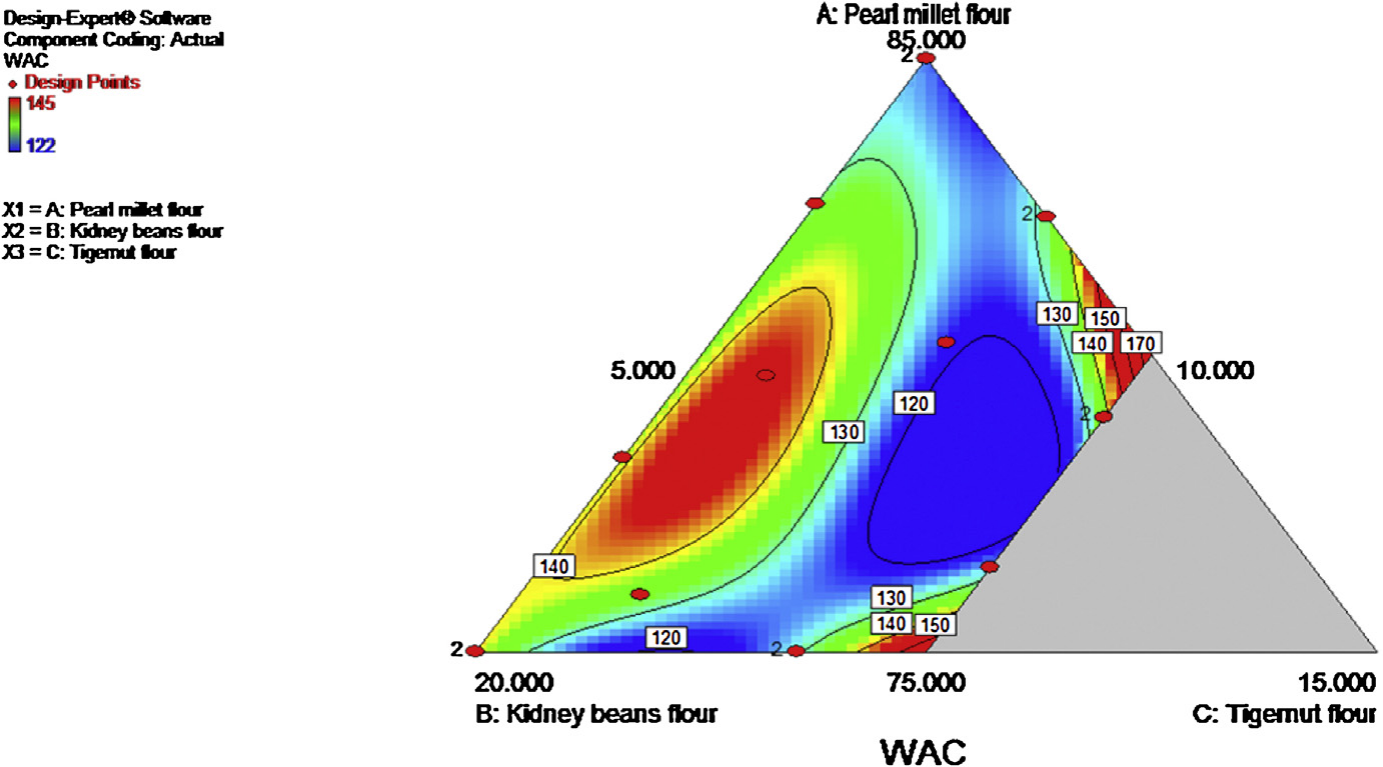
Contour plot showing the effect of pearl millet, kidney beans and tigernut composite flours on water absorption capacity.

The WAC ranged from 126–145%. Kidney beans significantly (p ≤ 0.05) increased the WAC. However, the ANOVA showed that the model (special quartic) and model terms (Linear mixture, AC, BC, A^2^BC, AB^2^C, ABC^2^) were significant (p ≤ 0.05). The R^2^ and adjusted R^2^ values were 0.9753 and 0.9470 respectively. The WAC values obtained were higher than the values obtained for Wheat flour, Wheat-plantain-tigernut composite flour (Bamigbola et al., 2016) and wheat-plantain flours (Mepba et al., 2007). Addition of legumes have been shown to increase WAC of composite flours (Awolu et al., 2015; Awolu et al., 2016b). High WAC is useful in product bulking and consistency. The contour plot showing the effect of the variables on the WAC is shown in Fig. 2.

**Fig. 3:**
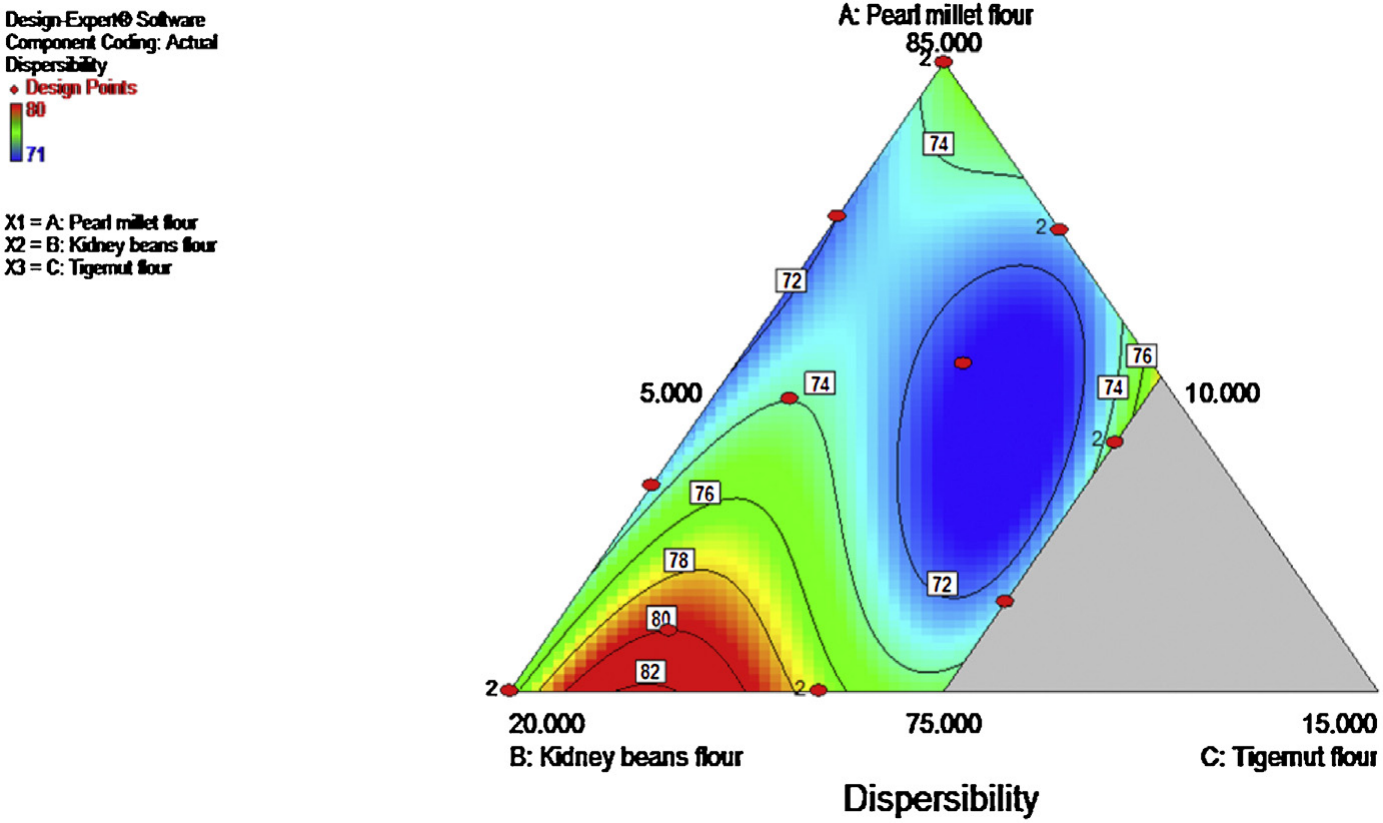
Contour plot showing the effect of pearl millet, kidney beans and tigernut composite flours on oil absorption capacity.

The result of the OAC of the composite flour ranged from 124% to 182%. High OAC products are better flavor retainer (Chandra and Samsher, 2013). The ANOVA showed that the model (special quartic) and model terms (Linear mixture, AB, AC, BC, ABC, AB(A-B), AC(A-C), BC(B-C)) were significant (p ≤ 0.05). The R^2^ and adjusted R^2^ values were 0.9947 and 0.9867 respectively. The ANOVA results indicate that all the independent variables enhanced the OAC. The p-value of the lack of fit was not significant (p ≤ 0.05). The contour plot (Fig. 3) showed that the pearl millet and kidney beans flours particularly promote the OAC.

The foaming capacity (0.96–2.95%) is very low. The combine effect of the raw materials must have been responsible for this. Bamigbola et al. (2016) reported foaming capacity of between 11 and 19% for wheat/plantain/tigernut composite flour while Mepba et al. (2007) reported foaming capacity of up to 26% for wheat/plantain composite flour. It does indicate that absence of wheat or presence of pearl millet flours was responsible for the low foaming capacity. The ANOVA results showed that only the model (special quartic) and model terms (AC, A^2^BC, AB^2^C) were significant (P ≤ 0.05).

**Fig. 4:**
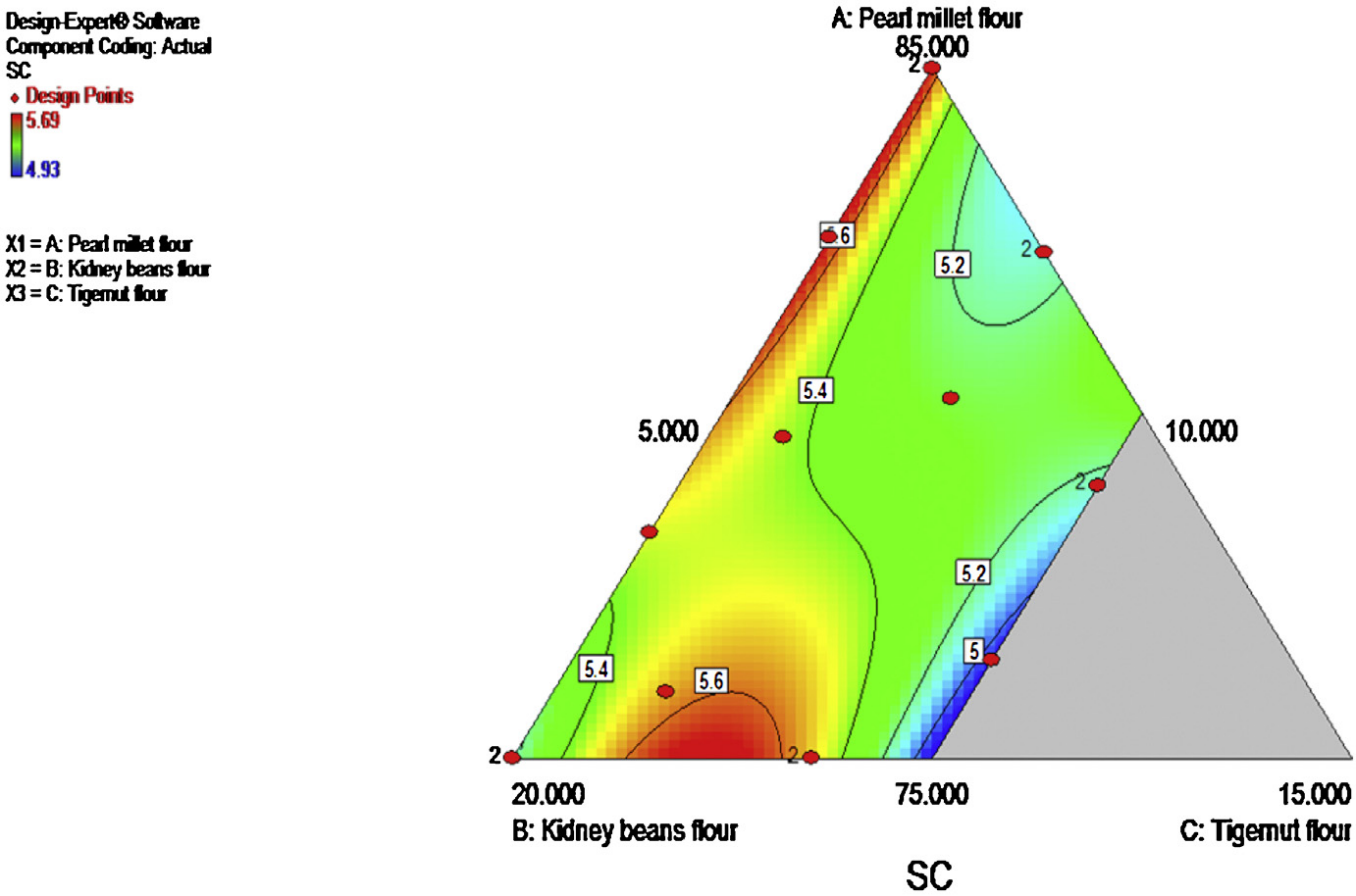
Contour plot showing the effect of pearl millet, kidney beans and tigernut composite flours on swelling capacity.

The swelling capacity ranged between 4.93 and 5.69 g/g. Pearl millet and kidney beans flours conspicuously increased the swelling capacity of composite flour as shown in Fig. 4. The model (cubic) and model terms (Linear mixture, AB, AC, BC, ABC, AC(A-C)) were significant (p ≤ 0.05). The R^2^ and the adjusted R^2^ values were 0.9881 and 0.9703 respectively.

### 3.2 Result of optimisation of the composite flours

Three best blends based on the optimization of functional properties were run 3 (75.956% pearl millet, 17.692% kidney beans, 6.352% tigernut flours), run 7 (85.000% pearl millet, 10.000% beans kidney, 5.000% tigernut flours), and run 13 (75.000% millet, 20.000% beans kidney, 5.000% tigernut flours). There are 38 optimization results. The choice is subjective, based on individuals objectives. In this case, the three best combinations selected from the 38 possible combinations were based on high or low WAC, high OAC, high foaming (≥ 1.70%) and high swelling capacity.

### 3.3 Pasting profile of the composite flour

The results of the pasting characteristics of the optimized composite flour samples (Runs 3, 7 and 13) and the control are presented in Table 2. The peak viscosity of the optimized composite flour samples ranged from 358–462 RVU. The peak viscosities of the composite flours were increased as the pearl millet content increased. The control (100% wheat flour) had very high peak viscosity value (1616 RVU). Peak viscosity is the maximum viscosity attained during or immediately after cooking is an indication of the pastes strength formed from gelatinization during processing (Adebowale et al., 2011; Maziya-Dixon et al., 2007). The peak viscosity values observed in this work is higher than values (199–222 RVU) obtained for toasted maize, soy beans and tigernut composite flours (Awolu et al., 2016b) but lower than values (2471–2864 RVU) obtained for wheat, amaranth, brewer spent grain and apple pomace composite flours (Awolu et al., 2016a). The high value in the later could be as a result of the addition of wheat flour and brewer spent grain (BSG) and apple pomace. Apple pomace and BSG added soluble fibres to composite flours which enhanced its viscoelastic properties.

The trough ranged from 354–442 RVU for the composite flour while it is 936 RVU for the control. The trend followed that of peak viscosity. Trough, which measures the ability of paste to withstand breakdown during cooling. The values obtained in this work are better than the values obtained by Awolu et al. (2016b) for toasted maize, soy bean and tigernut composite flours but lower than values obtained by Awolu et al., (2016a) for wheat, amaranth, brewer spent grain and apple pomace composite flours.

The breakdown viscosity had been shown to reflect the stability of the peak viscosity during processing (Moorthy, 1985). Starch with lower breakdown viscosity had been reported to possess higher capacity to withstand heating and shearing during cooking. The values obtained (3–20 RVU) were much lower to the values (5–81 RVU) obtained for toasted maize, soy beans and tigernut composite flours (Awolu et al., 2016b); 64–87 RVU obtained for wheat, plantain and tigernut flours (Bamigbola et al., 2016), and 845–1240 RVU obtained for wheat, amaranth grain, BSG and apple pomace flours (Awolu et al., 2016a). Runs 3 and 7 with lower breakdown viscosity could be due to high kidney bean (legume) contents in the composite flours. This same observation was observed by Awolu et al. (2016b) in composite flour consisting high soy beans (75% wheat, 20% soybean and 5% tigernut flours) where the breakdown viscosity was 5 RVU. In essence, the composite flour made from pearl millet, kidney beans and tigernut flours would have greater capacity to withstand heating and shearing, which could be advantageous in process handling of the composite flour.

The Final viscosity ranged from 782–940 RVU for the composite flour sample, while the final viscosity value for the control is 1906 RVU. Increased pearl millet content (as in run 7) had the increased the final viscosity of the composite flour. Final viscosity defines the particular quality of starch and stability of cooked paste. Lower final viscosity signifies reduced ability to form viscous pastes. The final viscosity obtained in this work is better than what were obtained by toasted maize-based composite flour (Awolu et al., 2016b) and wheat-plantain-tigernut flour composite flour (Bamigbola et al., 2016). Pearl millet could have enhanced the viscosities.

Pasting temperature was lowest in the control (100% wheat), closely followed by composite flour with 85% pearl millet (run 7). The sample with highest pearl millet flour (run 7) has consistently displayed better pasting characteristics, though, unlike 100% wheat flour. Pasting temperature is defined as the minimum temperature at which starch granules in the composite flour swells. In addition, run 7 had the best pasting time.

### 3.4 Results of rheological analysis of the composite flour

The results of mixolab of the composite flours (runs 7 and 13) are presented in Table 3. Mixolab measures dough development behaviour during mixing and heating, enabling the determination of protein and starch contribution to rheological characteristics of dough simultaneously (Rosell et al., 2007). There are five stages in Mixolab measurements.

Stage one (C1) is the dough development which corresponds to farinograph curve (Visitiu and Danciu, 2011). The water absorption for run 7 (85% pearl millet, 10% kidney beans and 5% tigernut flours) and run 13 (75% pearl millet, 20% kidney beans and 5% tigernut flours) were 51.3% and 46.3% respectively. Water absorption indicates the potential of protein molecules to absorb water, hence, an indicator of baking quality (Van Lill et al., 1995). In addition to protein content, starch, damaged starch, pentosans and gluten strength have been found to enhance water absorption of flours (Vizitiu and Danviu, 2011). The higher water absorption of run 7 could therefore be as a result of higher amounts of starch, damaged starch and pentosans in addition to protein in the sample. Run 13, though, had more kidney bean content had lower water absorption. This could be as a result of lower starch, damaged starch and pentosans. Pearl millet was higher in run 7, and could be the source of the higher starch, damaged starch and pentosans which resulted in the higher water absorption. Water absorption of 51.3% was reported for industrial wheat flour (Vizitiu and Danviu, 2011).

The dough development time (DDT) which is the time the dough have optimum viscoelastic properties for gas retention (Vizitiu and Danviu, 2011) were 19.97 and 26.22 mins for runs 7 and 13 respectively. Run 7, therefore, had a faster dough development time. The stability time for runs 7 and 13 were 9.18 and 1.57 mins respectively. The longer stability period is an indication of better protein quality. Since the stage one had been shown to correspond to farinograph curve, run 7 therefore had better dough quality for bread making than run 13.

In stage two, the protein network weakening rate (α) was −0.064 and −0.026 Nm/min for runs 7 and 13 respectively. In addition, the minimum consistency (C2), which is the minimum values of torque produced as the dough was subjected to mechanical and thermal stress were 0.43 and 2.73 Nm for runs 7 and 13 respectively. Run 13 therefore had a better resistance to thermal and mechanical stresses. A C2 of 0.36 Nm was reported for wheat cassava flour (Ibidapo *et al.,* 2015). Wheat flour has C2 of 0.4Nm. So run 7 has C2 value close to wheat flour. High C2 value has been reported to indicate good protein quality.

In the third stage (C3), the gelatinization rates (β) were 0.728 and 0.014 Nm/min for runs 7 and 13 respectively. The availability of dough heating and water from thermal denaturation of protein must have resulted in starch gelatinization (Thomas and Atwell, 1999). The maximum torques obtained in this stage were 0.83 and 2.92 Nm for runs 7 and 13 respectively. Run 7, therefore, had a higher gelatinization rate. It had been reported that modification of the physicochemical properties of the starch were the major operations in the last three stages (C3, C4, and C5) (Rosell et al., 2007).

In stage four (C4), the cooking stability (γ) were −0.532 and −0.018 Nm/min respectively. Run 13 had better cooking stability. The breakdown torques (difference between C3 and C4) were 0.70 and 0.20 Nm. The last stage (C5) is the setback stage (retrogradation of starch molecules), which resulted from decrease in temperature. The setback torque (difference between C5 and C4) were 2.4 Nm and 1.7 Nm in runs 7 and 13 respectively. Run 7 was therefore better shelf stable. Mixolab characteristics were better in run 7 than in run 13. The presence of pearl millet could account for this.

The Mixolab profiler indicating absorption, mixing index, gluten plus, viscosity, amylose and retrogradation for run 7 were one, six, four, one, nine and six respectively. Run 13 with 75% pearl millet had no result, indicating further that run 7 (85% pearl millet) sample had better Mixolab, hence, rheological behaviour. In comparison with 100% wheat flour, the results were absorption (2), mixing index (6), gluten plus (5), viscosity (8), amylose (6) and retrogradation (5) as reported by Mixolab Application Handbook (2012). Absorption represents water absorption and it has been reported to be influenced by the moisture content, protein content and the level of damaged starch in the flour. Mixing index indicated the overall protein quality; it shows the resistance of the flour to kneading. Addition of kidney bean must have accounted for the higher mixing index of the composite flour. The gluten + also measured the protein strength. Amylase represents resistance of starch to α-amylase with high value corresponding to low amylase activity. In this work, amylase value is 9 as against the value of 6 reported for 100% wheat flour. Retrogradation index defined the staling rate of the final product was six for the composite flour and five for 100% wheat flour.

## 4 Conclusion

Pearl millet-based composite flour offered a viable alternative to wheat-based composite flour for bread production. The results of the functional, pasting and rheological properties confirmed it. While the functional properties of pearl millet – based composite flour was better than 100% wheat flour, the 100% wheat flour had better pasting and rheological characteristics. On the other hand, pearl millet – kidney beans – tigernut composite flour (85%:10%:5%) had excellent rheological behaviour comparable with 100% wheat flour.

## Declarations

### Author contribution statement

Olugbenga O. Awolu: Conceived and designed the experiments; Performed the experiments; Analyzed and interpreted the data; Contributed reagents, materials, analysis tools or data.

### Competing interest statement

The authors declare no conflict of interest.

### Funding statement

This research did not receive any specific grant from funding agencies in the public, commercial, or not-for-profit sectors.

### Additional information

No additional information is available for this paper.

## Figures and Tables

**Table 1:** Functional properties of the composite flour.

S/N	A (%)	B (%)	C (%)	BD (g/ml)	Dispersibility (%)	WAC (%)	OAC (%)	FC (%)	SC (g/g)	SI (g/g)
1	82.33	10.00	7.67	0.81	72.00	132.00	140.00	2.70	5.15	0.07
2	85.00	10.00	5.00	0.80	75.00	125.00	155.00	1.87	5.65	0.06
3	75.96	17.69	6.35	0.76	80.00	135.00	175.00	1.90	5.56	0.06
4	82.55	12.45	5.00	1.03	72.00	129.00	182.00	1.89	5.69	0.06
5	75.00	16.44	8.57	0.78	76.00	130.00	164.00	1.88	5.45	0.05
6	76.42	13.58	10.00	0.75	73.00	126.00	148.00	1.62	4.93	0.06
7	85.00	10.00	5.00	0.81	75.00	126.00	155.00	1.70	5.69	0.06
8	75.00	16.44	8.57	0.79	78.00	128.00	168.00	1.83	5.58	0.06
9	78.27	16.73	5.00	0.69	73.00	140.00	61.00	1.77	5.48	0.06
10	79.65	14.46	5.90	0.73	74.00	145.00	171.00	0.96	5.43	0.06
11	82.33	10.00	7.67	0.83	74.00	133.00	140.00	2.95	5.15	0.08
12	78.95	11.05	10.00	0.79	76.00	138.00	132.00	1.89	5.16	0.06
13	75.00	20.00	5.00	0.76	75.00	138.00	124.00	2.76	5.15	0.06
14	78.95	11.05	10.00	0.78	74.00	138.00	125.00	1.88	5.16	0.06
15	75.00	20.00	5.00	0.75	75.00	140.00	124.00	1.89	5.19	0.04
16	80.21	12.17	7.62	1.03	71.00	122.00	112.00	1.89	5.26	0.04

*A − Pearl millet flour; B − Kidney beans flour; C − Tigernut flour; WAC − water absorption capacity; OAC − oil absorption capacity; FC − foaming capacity; SC − swelling capacity; SI − swelling index. **All values are means of triplicate samples. Values on vertical row with the same superscript are not significantly different (*p* < 0.05).

**Table 2:** Pasting profile of the flour.

Sample	PV (RVU)	Trough (RVU)	BV (RVU)	FV (RVU)	SB (RVU)	PT (min)	P. Temp (°C)
Run 3	390.00	387.00	3.0	840.00	461.00	7.00	91.25
Run 7	462.00	442.00	20.00	975.00	533.00	5.47	89.60
Run 13	358.00	354.00	4.00	782.00	428.00	6.67	90.45
Control	1616.00	936.00	680.00	1906.00	970.00	6.07	88.85

*PV − Peak viscosity, BV − Breakdown viscosity, FV − Final viscosity, SB − Setback, PT − Peak time, P. temp − Peak temperature. Run 3 = Pearl millet 75.956%, Kidney beans 17.692%, Tigernut 6.352%. Run 7 = Pear millet 85.000%, Kidney beans 10.000%, Tigernut 5.000%. Run 13 = Pearl millet 75.000%, Kidney beans 20.000%, Tigernut 5.000%. Control = 100% wheat flour.

**Table 3:** Pasting profile of the flour.

Parameter	Run 7	Run 13
Water absorption (%)	51.3	46.3
Dough Development Time (DDT)	19.97	26.22
Stability Time (min)	9.18	1.57
Protein networking, α (Nm/min)	−0.064	−0.026
Minimum constituency, C2 (Nm)	0.43	2.73
Gelatinization rate, β (Nm/min)	0.728	0.014
Maximum torques (Nm)	0.83	2.92
Cooking stability, γ (Nm/min)	−0.532	−0.018
Breakdown torque (Nm)	0.70	0.20
Setback torque (Nm)	2.4	1.7

## References

[bib0005] Adebowale A.A., Sanni L.O., Fadahunsi E.L. (2011). Functional and pasting properties of cassava-sweetpotato starch blends. Afr. J. Root Tuber Crop.

[bib0010] Adejuyitan J.A., Otunola E.T., Akande E.A., Bolarinwa I.F., Oladokun F.M. (2009). Some physicochemical properties of flour obtained from fermentation of tigernut (*Cyperus esculentus*) sourced from a market in Ogbomoso, Nigeria. Afr. J. Food Sci..

[bib0015] Ade-Omowaye B.I., Akinwande B.A., Bolarinwa I.F., Adebiyi A.O. (2008). Evaluation of tiger nut (Cyperusesculentus) −wheat composite flour and bread. Afr. J. Food Sci..

[bib0020] AOAC (2005). Official Methods of Analysis.

[bib0025] Akpata M.I., Akubor P.I. (1999). Chemical composition and selected functional properties of sweet orange (Citrus sinesis) seed flour. Plant Food Hum. Nutri..

[bib0030] Awolu O.O., Layokun S.K. (2013). Optimisation of two-steps transesterification production of biodisel from neem (*Azadirachta indica*) oil. Int. J. Energy Environ. Eng..

[bib0035] Awolu O.O., Obafaye R.O., Ayodele B.S. (2013). Optimization of solvent extraction of oil from neem (*Azadirachta indica*) and its characterizations. J. Sci. Res. Rep..

[bib0040] Awolu O.O., Oluwaferanmi P.M., Fafowora O.I., Oseyemi G.F. (2015). Optimization of the extrusion process for the production of ready-to-eat snack from rice, cassava and kersting’s groundnut composite flour. LWT Food Sci. Technol..

[bib0045] Awolu O.O., Osemeke R.O., Ifesan B.O.T. (2016). Antioxidant, functional and rheological properties of optimized composite flour, consisting wheat and amaranth seed, brewers’ spent grain and apple pomace. J. Food Sci. Technol..

[bib0050] Awolu O.O., Omoba O.S., Olawoye O.A., Dairo M. (2016). Optimization of production and quality evaluation of maize-based snack supplemented with soybean and tigernut (*Cyperus esculenta*) flour. Food Sci. Nutri..

[bib0055] Bamigbola Y.A., Awolu O.O., Oluwalana I.B. (2016). The effect of plantain and tigernut flours substitution on the antioxidant, physicochemical and pasting properties of wheat-based composite flour. Cogent Food Agric..

[bib0060] Chandra S., Samsher (2013). Assessment of Functional Properties of different flours. Acad. J..

[bib0065] Diniz A.M., Martin A.M. (1997). Optimization of nitrogen recovery in the enzymatic hydrolysis of dogfish (*Squalus acanthias*) protein: Composition of the hydrolysates. Int. J. Food Sci. Nutri..

[bib0070] Eke-Ejiofor (2015). Physico-chemical and pasting properties of starches from cassava, sweet potato and three leaf yam and their application in salad cream production. Int. J. Biotechnol. Food Sci..

[bib0075] Food and Agriculture Organization (FAO) (1995). Sorghum and Millets in Human Nutrition.

[bib0080] Giami S.Y., Bakebain O.A. (1992). Proximate composition and functional properties of raw and processed full fat fluten pumpkin (*Telfaria occidentalis*) seed flour. J. Sci. Food Agric..

[bib0085] Hassan H.A., Mustafa A.I., Ahmed A.R. (2013). Effect of incorporation of decorticated pigeon pea (*Cajanus cajan*) protein isolate on functional, baking and sensory characteristics of Wheat (*Triticum aestivum*) biscuit. Adv. J. Food Sci. Technol..

[bib0090] Jideani V.A. (2005). Characteristics of local pearl millet (*Pennisetum glaucum*) grains. Niger. Food J..

[bib0095] Kulkarni K.O., Kulkarni D.N., Ingle U.M. (1991). Sorghum malt based weaning food formulation preparation, functional properties and nutritive values. Food Nutri. Bull..

[bib0100] Manonmani D., Bhol S., Bosco S.J.D. (2014). Effect of red kidney bean (*Phaseolus vulgaris*) flour on bread quality. Open Access Libr. J..

[bib0105] Maziya-Dixon B., Dixon A.G.O., Adebowale A.A. (2007). Targeting different end uses of cassava: genotypic variations for cyanogenic potentials and pasting properties. Int. J. Food Sci. Technol..

[bib0110] Mepba H.D., Eboh L., Nwaojigwa S.U. (2007). Chemical composition: functional and baking properties of wheat-plantain composite flours. Afr. J. Food Agri. Nutri. Dev..

[bib0115] Moorthy S. (1985). Effect of different types of surfactants on cassava starch properties. J. Agric. Food Chem..

[bib0120] Mixolab Application Handbook (2012). Chopin Technologies.

[bib0125] Narayana K., Narsinga M.S. (1992). Functional properties of raw and heat-processed winged bean (P*sophocarpus Tertragonolobus*). Flour. J. Food Sci..

[bib0130] Oladele A.K., Aina J.O. (2007). Chemical composition and functional properties of flour produced from two varieties of tigernut (*Cyperus esculenta*). Afr. J. Biotechnol..

[bib0135] Pastukhov A., Dogan H. (2014). Studying of speed and temperature impacts on rheological properties of wheat flour dough using Mixolab. Agron. Res..

[bib0140] Rosell C.M., Collar C., Haros M. (2007). Assessment of hydrocolloid effects on the thermos-mechanical properties of wheat using. Mixolab. Food Hydrocol..

[bib0145] Saha S., Gupta A., Singh S.R.K., Bharti N., Singh K.P., Mahajan V., Gupta H.S. (2011). Compositional and varietal influence of finger millet flour on rheological properties of dough and quality of biscuit. LWT Food Sci. Technol..

[bib0150] Saleh A.S.M., Zhang Q., Chen J., Shen Q. (2013). Millet grains: nutritional quality, processing, and potential health benefits. Compr. Rev. Food Sci. Food Saf..

[bib0155] Thomas D.J., Atwell W.A. (1999). Starches, Eagan Press Handbook series.

[bib0160] Ukpabi U.J., Ndimele C. (1990). Evaluation of the quality of gari produced in Imo State. Niger. Food J..

[bib0165] Van Lill D., Purchase J.L., Smith M.F., Agenbag De Viliers G.A.O.T. (1995). Multivariate assessment of environmental effects on hard red winter wheat, I. Princile components analysis on yield and bread making characteristics. S. Afr. J. Plant Soil.

[bib0170] Vizitiu Daniel, Danciu Ioan (2011). Evaluation of farinograph and Mixolab for prediction of mixing properties of industrial wheat flour. Acta Universitatis Cibiniensis Series E: Food Technol..

